# Descriptive review of clinical data from 186 records of outpatients with IgA deficiency accompanied at a quaternary hospital in Brazil

**DOI:** 10.1186/1939-4551-8-S1-A162

**Published:** 2015-04-08

**Authors:** Fabiana Mascarenhas, Myrthes Toledo Barros, Leonardo Mendonça, Cristina Kokron, Karla Boufleur, Pablo Torres, Ana Karolina Barreto De Oliveira, Octavio Grecco, Jorge Kalil, Andrea Cohon

**Affiliations:** 1 Hcfmusp, Brazil; 2 Hospital Das Clínicas - Faculdade De Medicina – USP, Brazil; 3 University of São Paulo, Brazil; 4 Laboratory of Clinical Immunology and Allergy (LIM-60), University of São Paulo Medical School, Brazil; 5 Hospital Infantil Darcy Vargas, Brazil

## Background

IgA Deficiency (IgAD) is the most common immunoglobulin deficiency, with approximate incidence of 1 in 400 to 3000 individuals in general population. Selective IgA deficiency is defined as serum IgA levels less than 7 mg/dL with normal serum IgM and IgG levels. A subgroup of individuals, with more than 7 mg/dL but less than 30 mg/dL of serum IgA, are defined as partial IgA deficient patients (pIgAD). Many individuals with selective IgA deficiency are clinically asymptomatic. However, some patients have increased incidence of infections, mainly in respiratory and intestinal tracts, as well as presence of atopy, autoimmunity and cancer.

## Methods

Records review from children and adults outpatients followed at the Clinical Immunology and Allergy Division at Hospital das Clinicas of FMUSP. All patients were orally informed about the use of these clinical data. The objective was to report the clinical data observed in a cohort of IgAD patients followed between 1994 and 2014.

## Results

The most common associated condition found was atopic disease in about 60% of patients, specially rhinitis (in tIgAD and pIgAD). Prevalence of recurrent infection was 36%: 20% of sinusitis, otitis and amigdalitis, 10% of recurrent pneumonia (more than twice in the last year) and 18% of recurrent diarrhea. Autoimmunity condition was observed in 18% of the patients. Autoimmune diseases detected were thyroiditis, autoimmune hemolytic anemia, idiopathic thrombocytopenic purpura, rheumatoid arthritis, celiac-like disease, systemic lupus, Sjögren’s disease, juvenile rheumatoid arthritis, autoimmune hepatitis and vitiligo. The most prevalent disease observed was thyroid disease in about 13% of the patients. History of medication reaction was observed in 9% of them and contact dermititis in 8%. About 9% of the IgAD patients had malignancies or pre neoplastic conditions like MGUS (monoclonal gamopathy with uncertain signifcance). It was not possible to establish rate mortality among these patients because many patients lost follow up for unknown reasons, but so far, only one death was reported, and was attributed to sepsis.

## Conclusions

Total and partial IgAD has a broad spectrum of clinical associations and further investigations to immunological intervention may have clinical results.

